# Supplementary material for: Do verbal coaching cues and analogies affect motor skill performance in youth populations?
Source: PLoS One. 2023 Mar 2;18(3):e0280201. doi: 10.1371/journal.pone.0280201 (PMC9980803; doi:10.1371/journal.pone.0280201)
Supplement: S1 File — (DOCX) [file pone.0280201.s007.docx]

**Coaching cues (below) in correspondence with order schemes (top)**

As an example, if the participant drew scheme number 4 out of the random number generator, both the jump and sprint cues would be delivered in the following order:

D E C F B G A H J I

So, “sprint and focus on driving the ground back” (D) would be delivered first, “sprint as if you are a jet taking off into the sky ahead” (E) would be second, “sprint and focus on driving the ground back” (C) would be delivered third, and so on for all ten performances.

| **Scheme No.** | **Order Schemes** | | | | | | | | | |
| --- | --- | --- | --- | --- | --- | --- | --- | --- | --- | --- |
| Scheme 1 | A | B | J | C | I | D | H | E | G | F |
| Scheme 2 | B | C | A | D | J | E | I | F | H | G |
| Scheme 3 | C | D | B | E | A | F | J | G | I | H |
| Scheme 4 | D | E | C | F | B | G | A | H | J | I |
| Scheme 5 | E | F | D | G | C | H | B | I | A | J |
| Scheme 6 | F | G | E | H | D | I | C | J | B | A |
| Scheme 7 | G | H | F | I | E | J | D | A | C | B |
| Scheme 8 | H | I | G | J | F | A | E | B | D | C |
| Scheme 9 | I | J | H | A | G | B | F | C | E | D |
| Scheme 10 | J | A | I | B | H | C | G | D | F | E |

| **Jump cues** | |
| --- | --- |
| A = | “as you jump, focus on extending your legs” |
| B = | “as you jump, focus on extending your legs” |
| C = | "jump as high as you can" |
| D = | “jump as if the ground is suddenly hot and you have to get off it as quick as possible” |
| E = | “as you jump, focus on pushing the ground away” |
| F = | “as you jump, focus on pushing the ground away” |
| G = | "jump as high as you can" |
| H = | “jump as if the ground is suddenly hot and you have to get off it as quick as possible” |
| I = | “jump as if you are trying to catch a ball overhead at its highest point" |
| J = | “jump as if you are trying to catch a ball overhead at its highest point" |
| **Sprint cues** | |
| A = | “sprint as if you are being chased up a hill” |
| B = | “sprint as if you are a jet taking off into the sky ahead” |
| C = | “sprint and focus on driving the ground back” |
| D = | “sprint and focus on driving the ground back” |
| E = | “sprint as if you are a jet taking off into the sky ahead” |
| F = | "sprint as fast as you can" |
| G = | “sprint and focus on driving your legs back” |
| H = | “sprint and focus on driving your legs back” |
| I = | “sprint as if you are being chased up a hill” |
| J = | "sprint as fast as you can" |
