# Supplementary material for: Do verbal coaching cues and analogies affect motor skill performance in youth populations?
Source: PLoS One. 2023 Mar 2;18(3):e0280201. doi: 10.1371/journal.pone.0280201 (PMC9980803; doi:10.1371/journal.pone.0280201)
Supplement: S2 File — (DOCX) [file pone.0280201.s008.docx]

**Jump cues (not delivered in the below order)**

**French translation version**

| 1. "Sautez le plus haut possible" |
| --- |
| 1. “Gardez les genoux en extension pendant le saut” |
| 1. “Pendant que vous sautez, Essayez de faire un impulsion forte avec le sol” |
| 1. “Minimsez au maximum le temps de contact avec le sol pendant le saut |
| 1. "sautez comme si vous essayiez d'attraper une balle au-dessus de votre tête à son point le plus haut" |

**Jump cues (not delivered in the below order)**

**Persian translation version**

| 1. " اقفز عاليا بقدر ما تستطيع" |
| --- |
| 1. “حافظ على ركبتيك ممدودتين أثناء القفز” |
| 1. "عندما تقفز ، ركز على دفع الأرض بعيدًا" |
| 1. "أثناء القفز ، حاول تقليل وقت التلامس مع الأرض قدر الإمكان" |
| 1. “"القفز كما لو كنت تحاول الإمساك بالكرة في أعلى نقطة لها" |

**Sprint cues (not delivered in the below order)**

**French translation version**

| 1. "Courir le plus vite possible" |
| --- |
| 1. “Courez et greffer le sol en arrière” |
| 1. “Snchroniser vos jambs pendant la course” |
| 1. "sprintez comme si vous étiez un avion décollant vers le ciel devant vous" |
| 1. “sprintez comme si vous étiez poursuivi en haut d'une colline » |

**Sprint cues (not delivered in the below order)**

**Persian translation version**

| 1. "العدو بأسرع ما يمكن" |
| --- |
| 1. "الركض والتركيز على قيادة الأرض للخلف" |
| 1. “اركض بسرعة وركز على قيادة ساقيك للخلف " |
| 1. "انطلق بسرعة وكأنك طائرة تقلع في السماء أمامنا" |
| 1. “اركض كما لو كنت مطاردًا إلى أعلى التل " |
