# Supplementary figures and images for: Do verbal coaching cues and analogies affect motor skill performance in youth populations?
Source: PLoS One. 2023 Mar 2;18(3):e0280201. doi: 10.1371/journal.pone.0280201 (PMC9980803; doi:10.1371/journal.pone.0280201)

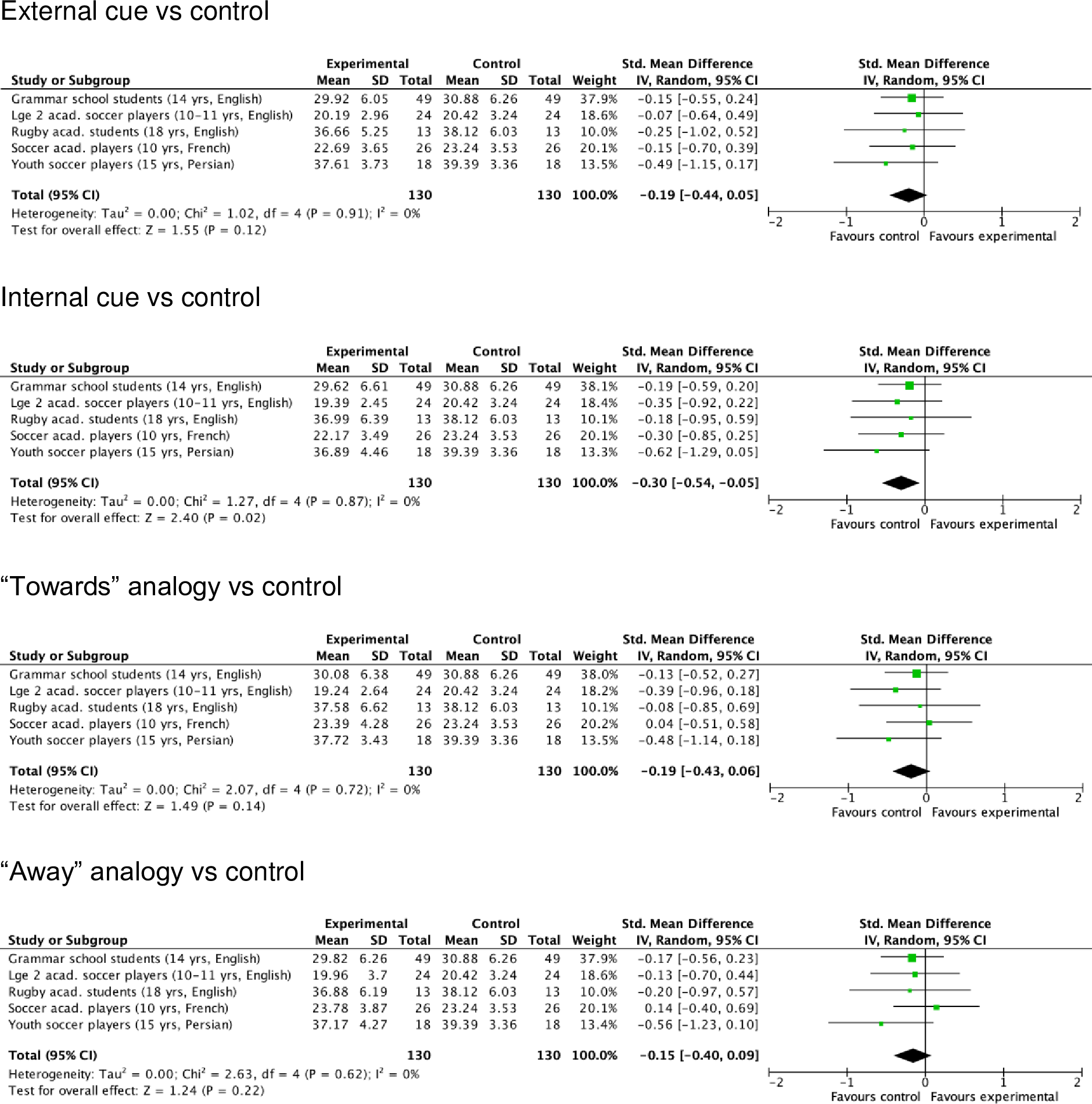

Supplement: S1 Fig — (TIF) [file pone.0280201.s009.tif]

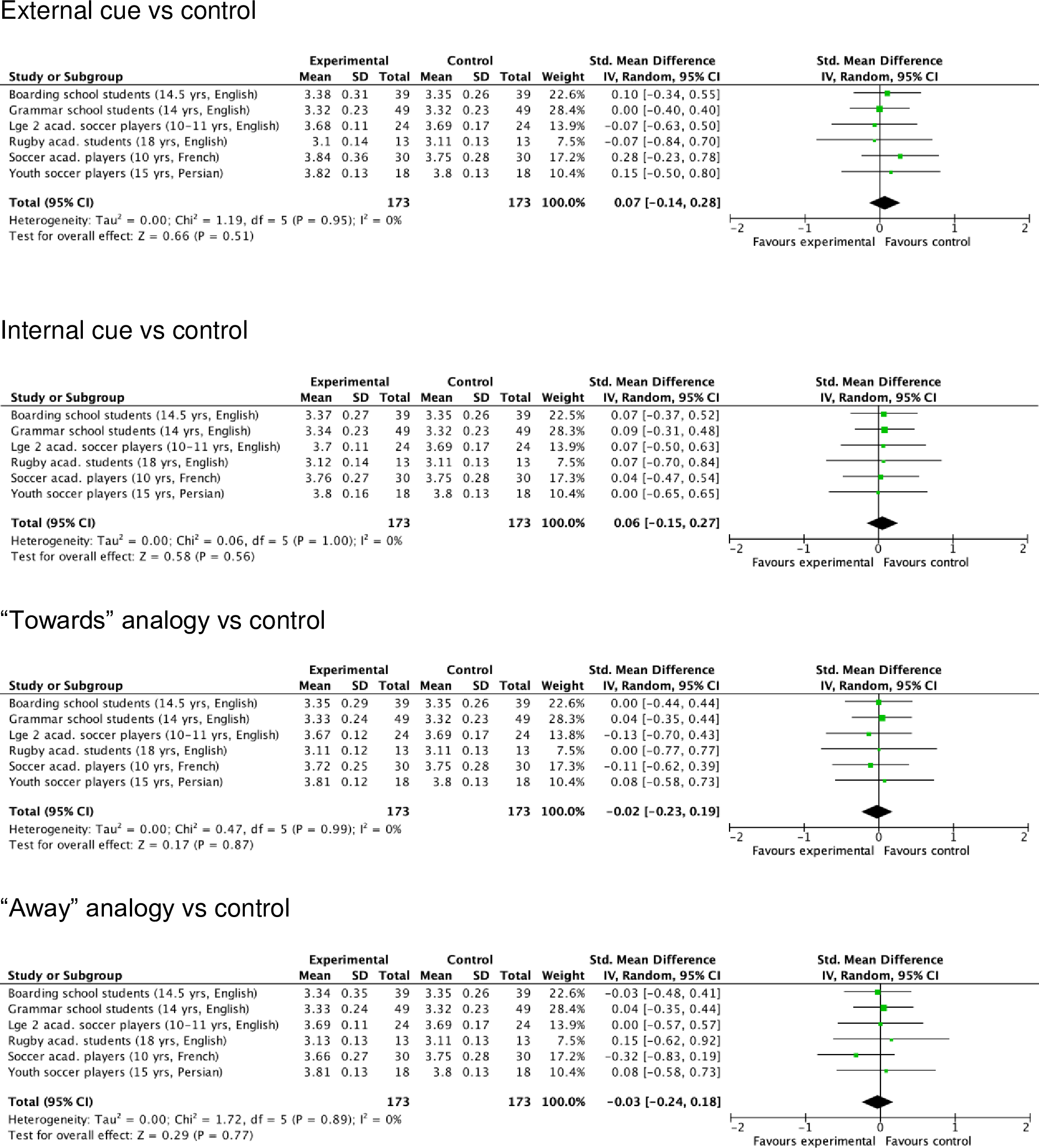

Supplement: S2 Fig — (TIF) [file pone.0280201.s010.tif]
