# Supplementary material for: Do verbal coaching cues and analogies affect motor skill performance in youth populations?
Source: PLoS One. 2023 Mar 2;18(3):e0280201. doi: 10.1371/journal.pone.0280201 (PMC9980803; doi:10.1371/journal.pone.0280201)
Supplement: Attachment — Submitted filename: PONE-D-22-21775-v1.doc [file pone.0280201.s011.doc]

**Model for Manuscript Review**

**Research articles**

**Manuscript title:**

PONE-D-22-21775 - Do verbal coaching cues and analogies affect motor skill performance in youth populations?

| **Títle** |  |
| --- | --- |
| Is it understandable and concise? | ( x ) Yes ( ) Not |
| Reflects the content? | ( x ) Yes ( ) Not |
| **Abstract** |  |
| It includes: objectives, methodology, key findings and conclusions? | ( ) Yes ( x ) Not |
| **Introduccion** |  |
| The investigation was carried out in a suitable theoretical structure? | ( ) Yes ( x ) Not |
| Clear leaves the questions you want to answer and objectives of the work? | ( ) Yes ( x ) Not |
| The cited references are current and relevant? | ( ) Yes ( x ) Not |
| **Methods** |  |
| The methods presented are appropriate to achieve the proposed objectives? | ( ) Yes ( x ) Not |
| The selection and composition of the sample are adequately described? | ( ) Yes ( x ) Not |
| The data collection process and the tools used are described clearly? | ( ) Yes ( x ) Not |
| The statistical analysis and the research design appropriate? | ( x ) Yes ( ) Not |
| **Results** |  |
| The presentation of the results clear? | ( ) Yes ( x ) Not |
| The main results are highlighted without the inclusion of interpretation and comparisons? | ( ) Yes ( x ) Not |
| The results evaluate the proposed objectives? | ( ) Yes ( x ) Not |
| Tables and figures are properly numbered, labeled and explained? | ( ) Yes ( x ) Not |
| **Discussion and Conclusion** |  |
| The results are discussed based on the literature? | ( x ) Yes ( ) Not |
| Author's interpretations show the safety and soundness? | ( x ) Yes ( ) Not |
| The limitations of the work are presented? | ( x ) Yes ( ) Not |
| The conclusions of the study are presented? | ( x ) Yes ( ) Not |
| The conclusions respond to the objectives? | ( x ) Yes ( ) Not |

**General comments:**

**Title**

Are presented satisfactorily.

**Abstract**

It is written in a structured way, however, the methodology is written in a very summarized way which ends up making the findings and conclusions of the article.

There is no description of the methodology in the abstract that would allow a better understanding of what was researched.

The conclusions are very timid, making the main findings difficult.

Please confirm that the Keywords are listed as descriptors in health sciences.

**Introduction**

This is very extensive, and on the other hand methodologically explains some points that should this in methodology and not in the introduction. The introduction is not starting from general to specific. It should initially present a more general approach and gradually address the problem (gap) and then present the objective.

The introduction should be more focused on the construct and not on the methodology of what is being researched.

Mentioning that there are few studies or that research is scarce does not seem to me to be a robust justification for the study. please review this.

**Methods**

It should present more clearly the design of the study. A CONSORT or time line should be presented in order to get a better view of the study design.

The sample should be better explained with the number of subjects presented initially and then present the inclusion and exclusion criteria.

**Results**

I consider that the results were very simplistic and practically do not clarify what was proposed. I suggest that this topic be further explored in order to better support the manuscript.

**Discussion**

It should reaffirm the objectives and start discussing the results in the chronological order that appear in the item results.

**Conclusion**

Are presented satisfactorily.

**References**

Please confirm the formatting of the references and of the 37 references 19 are current and 18 are more than five years old. It is suggested that the references be updated.

**Overview**

The manuscript presented addresses a relevant research topic.

It would be advisable to do a general review.

**Specific comments and suggestions:**

**Outcome evaluation**

- Accept unchanged	(	)
- Accepted with minor changes	(	)
- Accepted with major changes	( x )
- Rejected	(	)
