# Supplementary material for: Do verbal coaching cues and analogies affect motor skill performance in youth populations?
Source: PLoS One. 2023 Mar 2;18(3):e0280201. doi: 10.1371/journal.pone.0280201 (PMC9980803; doi:10.1371/journal.pone.0280201)
Supplement: Attachment — Submitted filename: Response to Reviewers.docx [file pone.0280201.s012.docx]

**We would like to sincerely thank both reviewers and editor for their time and efforts in helping to improve our manuscript. We feel the recommended changes have improved the paper, particularly in relation to its scientific rigour and replicability. Below, is an itemised response to each of the points raised and we hope that this brings the paper up to the high standard required for publication in PLOS One. In some cases, both reviewers requested similar changes and these were relatively simple to implement. In others, there were instructions that somewhat contradicted each other (for example the results section!) so we did our very best to come the most appropriate compromise between the recommendations of each reviewer. In places, we have subdivided down the reviewers’ comments into smaller points so as to address the specific aspects of each of the comments. We hope that in all cases that our responses reflect the requests of the reviewers and we once again thank them for their valuable input.**

**COMMENTS FOR REVIEWER #1**

**General comments:**

**Reviewer: Title**

Are presented satisfactorily.

**Response:** We appreciate this comment.

**Reviewer: Abstract**

It is written in a structured way, however, the methodology is written in a very summarized way which ends up making the findings and conclusions of the article. There is no description of the methodology in the abstract that would allow a better understanding of what was researched.

**Response**: We appreciate that our description of the methods was ambiguous here so we added more detail to specifically reflect the approach we took in a clearer way:

*“Across several international locations, we undertook a series of separate experiments to determine the effect of external cues (EC), internal cues (IC), analogies with a directional component (ADC) and control cues on sprint time (20 m) and vertical jump height in youth performers. For greater statistical power, we combined these data using internal meta-analytical techniques to pool results across each test location. We amalgamated this approach with a repeated measures analysis to determine if there were any differences between the ECs, ICs and ADCs within the different experiments.”*

Because we have used a novel statistical approach to pool data we felt the description of the methods needed to heavily reflect the statistical approach taken. To indicate to the reader the general objective we wrote:

“*Across several international locations, we undertook a series of separate experiments to determine the effect of external cues (EC), internal cues (IC), analogies with a directional component (ADC) and control cues on sprint time (20 m) and vertical jump height in youth performers.”*

We would also hope that the state purpose of the research would also give the reader some insight into the reason we undertook it. However, in response to the reviewer’s comment we also slightly edited the ‘purpose’ section of the abstract:

*“The way coaching instructions are worded to performers can impact on the quality with which a subsequent motor skill is executed. However, there have been few investigations on the effect of coaching instructions on basic motor skill performance in youths.”*

**Reviewer**: The conclusions are very timid, making the main findings difficult.

**Response**: Given that our results were somewhat controversial in this area, we wanted to be equivocal about their implications for youths. However, we believe the reviewer is correct and we should not shy away from our otherwise interesting findings. Accordingly, we have now written:

*“These results suggest that the type of cue or analogy provided to a youth performer has little subsequent effect on sprint or jump performance. Accordingly, coaches might take a more specific approach that is suited to the level or preferences of a particular individual.”*

**Reviewer**: Please confirm that the Keywords are listed as descriptors in health sciences.

**Response:** We have now given the keywords a more health-orientated focus:

*“Fundamental movement skills, instructions, run, jump, youth”*

**Introduction**

**Reviewer**: This is very extensive, and on the other hand methodologically explains some points that should this in methodology and not in the introduction.

**Response**: Please see corresponding answer below for explanation on this particular request by the reviewer.

**Reviewer**: The introduction is not starting from general to specific. It should initially present a more general approach and gradually address the problem (gap) and then present the objective.

**Response**: We agree with the reviewer that we were too quick to get into the main details of the article without first explaining to readers about the underpinning theory and giving some relevant background. We have now rearranged the introduction to first explain what internal and external cues are and then to relate them to the constrained action hypothesis which is the general theory that underpins research of this type. Only after this do we delve into the relevant research and the related gaps that exist:

*“Coaching instructions that direct a performer’s attention externally (i.e. a focus placed outside of the body) or internally (i.e. a focus on body part movement) have been shown to have demonstrable effects on subsequent motor skill performance (1–3). The constrained action hypothesis (6) suggests that an external focus of attention can result in improved motor performance by increasing the automaticity of movement control during a given action, also supporting implicit learning (7).”*

We have also moved the evidence cited by Winkelman towards the beginning of the introduction as this was more general in nature and was better placed earlier in the work to set the scene for the reader, as per the reviewer’s request.

**Reviewer**: The introduction should be more focused on the construct and not on the methodology of what is being researched.

**Response**: With the changes we have now made, we hope that this particular request of the reviewer has been addressed in the process. As per our answer about the methods section above, we believe a certain amount of information on this was required in the introduction section as some of the concepts have not been studied before and could be unfamiliar even to researchers and practitioners who are very familiar with the literature in this area. As our study is the first of its kind in this population, and with the described methods, we believe some relevant background information was necessary to provide early in the piece. We hope the reviewer can appreciate our approach on this.

**Reviewer**: Mentioning that there are few studies or that research is scarce does not seem to me to be a robust justification for the study. please review this.

**Response**: Thank you for this observation – we agree that more justification for the study is required. In addition to the changes detailed below, we believe that in too many places we highlighted the lack of research in this area and we have now reduced this in line with the reviewer’s request. We have now added a sentence which justifies exactly why this scarce research has negative implications for coaching and learning in a practical context. We hope this adds further justification as to why the low amount of research in this area has further effects on coaching and learning:

*“To date, there have been very few investigations on the effect of various different foci of attention on the performance or retention of basic FMS, such as running and jumping, in youths. This has made it particularly difficult to determine whether or not the manipulation of attentional focus can enhance performance and subsequent motor learning in young individuals. Accordingly, coaches and teachers may therefore not be using optimal methods when attempting to drive motor learning in these populations.”*

We would also direct the reviewer to the pre-existing text that is below this additional text which also describes the problems associated with the low amount of studies in this area:

*“Two systematic reviews (6,7) have provided a comprehensive overview of the various studies that have been undertaken in this area, however, of the investigations carried out in children, most related to sport-specific skills such as basketball dribbling, golf putting and tennis serving, as opposed to basic FMS that might be taught in physical education, such as jumping. Moreover, not a single study in children has examined the effect of different attentional foci on the execution of running or sprinting, a vital FMS for children as they develop. This is particularly concerning given that physical literacy has been associated with higher levels of physical activity (8) meaning that if youths can achieve mastery in FMS such as running, they may be more likely to engage in physical activity across the lifespan (9). Accordingly, the teaching of these skills in a way that best resonates with the target population appears to be a vital component of physical education provision.”*

Further down in the introduction, we have also added more justification alongside the initial observation that research in the area is scarce:

*“Moreover, to our knowledge, no previous study has examined these types of foci when combined with analogies in any population, let alone in youths; this despite the merging of ECs with analogies being suggested to be an effective way to retain a distal focus for optimal performance (1,4). This could have important implications for coaching and learning as the combination of ECs and analogies could represent a new tool for coaches that can enhance the contextual understanding of a performer and, by extension, the learning of key movement skills.”*

**Methods**

**Reviewer**: It should present more clearly the design of the study. A CONSORT or time line should be presented in order to get a better view of the study design.

**Response**: We have now included a diagram to depict the research design of the study. We have included it as Figure 1 in the text.

**Reviewer**: The sample should be better explained with the number of subjects presented initially and then present the inclusion and exclusion criteria.

**Response**: We have now added further information to this effect in the ‘Experimental design’ section of the methods:

*“We recruited 173 participants from a variety of different backgrounds, developmental levels and ages. The descriptive characteristics of the various groups can be viewed in Table 1. Only individuals under the age of 18 were eligible to take part and though the study was open to female participants, we were unable to recruit any into the various cohorts. Only healthy individuals were considered and the study was open to both trained and untrained participants.”*

**Results**

**Reviewer**: I consider that the results were very simplistic and practically do not clarify what was proposed. I suggest that this topic be further explored in order to better support the manuscript.

**Response**: We were initially restricted by space for figures and tables as indicated in the author guidelines and so we had to include the forest plots (which contain the relevant information) in the supplemental material. However, in line with the reviewer’s request, we have now added further information to the results section. The most important change relates to the internal meta-analytical findings. For example, the reader was previously referred to the supplemental information to view the forest plots, however, they no longer need to do this as we have summarized the results in the text:

*“In summary, there were no significant differences between the control cue and coaching instructions in any of the analyses. Effect sizes, representing the differences between performance under the various instructions, ranged from -0.03 (95% confidence interval: -0.24, 0.18) to 0.07 (95% confidence interval: -0.14, 0.28) for the sprint measures and were all classified as ‘trivial’. Effect sizes for the jump measures ranged from -0.30 (95% confidence interval: -0.54, -0.05) to -0.15 (95% confidence interval: -0.40, 0.09). Across all analyses, the only effect size that crossed the threshold from ‘trivial’ to ‘small’ was that for the IC (“as you jump, focus on extending your legs”) when compared to the control cue ("jump as high as you can") in the jump analysis. In that instance, there was a ‘small’ statistically significant effect size which favoured the control cue (d = -0.30, 95% confidence interval: [-0.54, -0.05], p=0.02).”*

In relation to the above, we previously had only reported one effect size that had crossed the threshold from ‘trivial’ to ‘small’, however, we had failed to indicate whether this effect size related to the jump analysis or to the sprint analysis. We have now made this clear in the text. We have also added a p-value for this statistic. To help the readers to understand this result, we have also added in brackets, the coaching cues that relate to the particular results (i.e. “as you jump, focus on extending your legs”) so that context is provided while reading this section.

In relation to the second paragraph of the results which relates to the repeated measures analysis, we left this as it was because all relevant p-values and statistics are already contained in Table 3 which is in the text and is not supplementary material. In the interests of avoiding repetition, we refer the reader to the table instead of repeating the results in the text. We hope the reviewer can appreciate our rationale for this decision. We have, however, clarified and edited some of the text in this paragraph to demonstrate the results to the reader in a more understandable way.

**Discussion**

**Reviewer**: It should reaffirm the objectives and start discussing the results in the chronological order that appear in the item results.

**Response**: We have now started the discussion by saying:

*“The purpose of this research was to determine the effectiveness of ICs, ECs and ADCs on motor skill performance in youths in various different populations, ranging from school children to academy athletes, and across a variety of international contexts and languages. We hypothesised that ECs, ICs and ADCs would be more effective than control cues for increasing jump and sprint performance, that ECs would be more effective than ICs and that ADCs would be more effective than both ECs and ICs.”*

We had initially stated the results as they appeared in the results section but we appreciate that this may not have been clear enough and we have added some additional wording at the start of the discussion to differentiate the results of the two main analyses. As requested by the reviewer, the meta-analytical results are discussed first in the discussion as they are listed first in the results and the repeated measures second.

**Conclusion**

**Reviewer**: Are presented satisfactorily.

**Response**: We would like to thank the reviewer for this comment.

**References**

**Reviewer**: Please confirm the formatting of the references and of the 37 references 19 are current and 18 are more than five years old. It is suggested that the references be updated.

**Reviewer**: The four main academic co-authors of this article wrote it based on what we saw as the most relevant articles on this topic. Studies such as those carried out by Wulf, Porter and Winkelman are older pieces of work but were some of the formative investigations in this área and we feel it is very necessary to retain them in our own study, despite their advancing age. We did review the reference list but feel it is appropriate and that it might be difficult to construct the paper in the way that we have if we were to change the composition of the references. We hope the reviewer can appreciate our view on this particular issue.

**COMMENTS FOR REVIEWER #2**

**We would like to sincerely thank both reviewers and editor for their time and efforts in helping to improve our manuscript. We feel the recommended changes have improved the paper, particularly in relation to its scientific rigour and replicability. Below, is an itemised response to each of the points raised and we hope that this brings the paper up to the high standard required for publication in PLOS One. In some cases, both reviewers requested similar changes and these were relatively simple to implement. In others, there were instructions that somewhat contradicted each other (for example the results section!) so we did our very best to come the most appropriate compromise between the recommendations of each reviewer. In places, we have subdivided down the reviewers’ comments into smaller points so as to address the specific aspects of each of the comments. We hope that in all cases that our responses reflect the requests of the reviewers and we once again thank them for their valuable input.**

**Reviewer**: The authors have written a manuscript investigating cues of external (EC) or internal (IC) focus, with an additional condition of analogies with a directional component (ADC) on sprint and jump performance in youth performers. A control cue condition was also included. The authors have designed an interesting study of the attentional focus/cueing literature. However, it is recommended that the authors reorganize this original manuscript to improve the clarity and understanding of both the findings and rationale for the study. Below are general comments for the author's consideration.

**Response**: Many thanks for these comments. We have made an itemised response to these comments below.

**General Comments (GC):**

**Reviewer**: GC1: There are no lines or pages provided so specific comments will attempt to direct the author by sections, paragraphs, and sentence order. For future submissions, authors should consider including page and line numbers.

**Response**: This was an oversight by us but we have now added page and line numbers so that any further changes can be more accurately indicated. We appreciate the reviewer’s patience on this matter.

**Reviewer**: GC2: Authors are encouraged to include what measures were chosen to indicate ‘performance’ and ‘retention’ in the abstract. Currently, it is ambiguous and the abstract does not indicate what is measured, i.e. sprint performance (time, velocity, etc) and jump performance (distance/height, etc).

**Response**: We have removed the word ‘retention’ from the abstract as this was not specifically measured. We have also added edited the wording to reflect the specific measures used:

*“Across several international locations, we undertook a series of separate experiments to determine the effect of external cues (EC), internal cues (IC), analogies with a directional component (ADC) and control cues on sprint time (20 m) and vertical jump height in youth performers.”*

**Reviewer**: GC3: Many of the statements are written colloquially. The authors are encouraged to stick to an objective form of writing and reduce any colloquial/subjective overtones.

**Response**: We have done several general reviews of the manuscript to amend this issue. We now hope that the paper reads in a more objective manner.

**Reviewer**: GC4: The authors are encouraged to either choose ‘attentional focus’ or ‘cues’ as the nomenclature for delivering relevant information. Alternatively, discuss how they are related. It is true that cues can direct the focus of an athlete, but the reader needs to have background knowledge of what a cue is. Currently, the author introduces attentional focus and then switches to the nomenclature of cues. Similarly, ‘instructions’ are also discussed. Authors are encouraged to choose either ‘cues’, ‘attentional focus’, or ‘instructions’.

**Response**: In line with the reviewer’s suggestion, we have decided that the most appropriate option is to use the term ‘coaching cue’ throughout the work as this is used most commonly in the literature and was the term that we asked the participants to focus on in each experiment (i.e. “Focus as hard as you can on this cue during the jump”). In places we had used these terms interchangeably and we agree with the reviewer that this is not correct. In limited places we have elected to retain some of the original wording but only in an attempt to contextualise the use of ‘coaching instructions’ ‘attentional focus’. They are no longer used interchangeably in the text and we are now firmly using the term ‘cue’.

**Reviewer**: GC5: A concern of neuromuscular fatigue for these athletes is worrying. Each participant spent about 25 to 30 minutes performing either a 20-m sprint or “jump”. Is there any evidence to support that 10 repetitions of 20-metre sprints and jumps would not be affected by neurological fatigue? Particularly populations are not homogeneous in their training experience.

**Response**: We were also aware that fatigue could have been a factor and this is why we took several controls to prevent its effects. Of course, in any environment, fatigue cannot be entirely minimised or discounted but we undertook the following stringent measures to prevent its effects on our results (all are detailed in the methods section of the paper with reference to offsetting fatigue and order effects):

Most importantly, we incorporated specific study design elements to reduce the order effects of fatigue. We detail this in the methods section entitled ‘coaching cues’:

*“A Latin square design was used to offset order effects due to fatigue or other factors that could impact upon participants’ performance. Each participant was randomly allocated a specific “order scheme” (between 1 and 10) via a random number generator (https://www.random.org/). This ‘order scheme’ determined the sequence in which each individual received their instructional cues prior to jumping or sprinting with each letter corresponding to a particular coaching cue, which can be seen in the supplemental information. As participants sprinted and jumped twice for each cue, each cue appeared twice. The order schemes can also be seen in the supplemental information.”*

If the reviewer refers to the supplemental information file ‘Suppl info - Cue order schemes’ they will see more information on this. In short, each participant did the sprints and jumps in different randomised orders. For example, because these orders were randomised, Participant “A” could have done the control cue fifth while Participant “B” may have done it seventh, or ninth etc etc. Simialrly, Participant “A” could have done the external cue second while Participant “B” could have done it seventh. On paper this seems rather simple but in practice was extremely challenging as each participant had to be allocated an order scheme and given their cues in a different order corresponding to that particular scheme. In the end, very few participants will have done the sprints in the same order because they were all allocated different order schemes meaning the effects of fatigue will have been mostly minimised.

In addition to the above, we took the usual measures one would be expected to do to allow recovery of the creatine phosphate system between short quick bursts of action. In this way, every sprint and jump was theoretically a fully rested new effort done in a primed and freshened state. The following studies support our approach in minimising fatigue in this way:

**Grant et al. (2011, JSCR)** showed that “*a 1:10 sprint-to-rest ratio allows full performance recovery between 15-m sprints*”. However, in our work we used a 20 m distance but we also utilised a 1:40 sprint to rest ratio to ensure maximal recovery.

**Read et al. (2001, JSCR)** showed that “*a 15-second rest interval was sufficient for recovery during the performance of depth jumps*”. However, in our work we provided x8 times this amount with at least a two minute rest between jumps of a lower intensity.

**Woolf et al (2018, ACSM)** showed that 60 secs was also sufficient between efforts.

We hope the above provisions reassure the reviewer that we took utmost care to ensure that data were collected in a rigourous manner.

Specific Comments:

**Introduction**

**Reviewer**: Paragraph 1, Sentence 1: Authors are encouraged to change the wording so it is unique when compared to the abstract.

**Response**: This has now been edited substantially due to this and other recommendations from Reviewer #1.

**Reviewer**: Paragraph 1: The paragraph seems rushed, without defining or describing concepts properly. It begins with talking about ‘learning’ and then finishes with discussing ‘performance’. From a motor control perspective, these two concepts are noticeably different. Similarly, although the constrained action hypothesis outcome is described, it is unclear how it underpins the automaticity of movement. The authors are encouraged to describe the importance of each of these concepts and how they are related. Currently, the reader needs to be aware of attentional focus (AF) literature to make sense of the paragraph.

**Response**: We agree with the reviewer and, indeed, Reviewer #1 also made similar comments. In line with both, we have now begun the introduction in a more general way, looking to become more specific as it continues. Now, instead of proceeding straight into potentially abstract topics for a reader, we start with a definition of a coaching cue followed by an explanation of the constrained action hypothesis and its mechanisms as a sound basis for later expansion in the introduction. To achieve this we have added additional words on mechanisms from Kal et al. (2013):

*“A coaching cue is a verbal instruction that can be used to focus an individual’s attention on a movement so as to optimise its execution (1). Cues that direct a performer’s attention externally (i.e. a focus placed outside of the body) or internally (i.e. a focus on body part movement) have been shown to have demonstrable effects on subsequent motor skill performance (2–4). The constrained action hypothesis (5) suggests that an external focus of attention can result in improved motor performance by increasing the automaticity of movement control during a given action, also supporting implicit learning (6). It has been proposed that an external focus reduces the attentional capacity that is needed to carry out a movement which can also be enhanced through greater coordination between working muscles (7). Accordingly, the manner in which coaching cues are worded and presented to performers can immediately impact on the quality with which motor skills are executed (8) and in the longer term, this can be reinforced through the learning process (9).*

**Reviewer**: Paragraph 2: The paragraph needs to be rewritten. The authors being concerned about the lack of research on how AF can affect fundamental movement skills (FMS) is an opinion and not objective. Stick to the facts, and report what the literature supports and what are the gaps. Refrain from including any opinions.

**Response**: Agreed. We have now removed the references to FMS and have referred only to the specific skills on which the research related to. We have made various edits throughout in accordance with this request from the reviewer.

**Reviewer**: Furthermore, it could be argued that sport-specific skills require more physical literacy than FMS, as FMS are gross-motor tasks whereas sport-specific skills require fine-motor coordination, thus performers need to be more physically literate to perform sport-specific tasks.

**Response**: In line with the previously mentioned change, this has now been edited/removed from the manuscript to avoid any confusion and to be more accurate.

**Reviewer**: Lastly, without supporting evidence that physical education (PE) should be teaching FMS over sport-specific skills, this comes across as a subjective perspective of the author. PE classes should be progressing into sport-specific skills after FMS have been taught, yet it could be true that FMS should not be overlooked.

**Response**: Agreed, and we have now removed the reference to physical education in line with this recommendation. In line with the previous two requests, we have made the necessary edits to maintain clarity and coherence with surrounding text.

**Reviewer**: Paragraph 3, Sentence 1: Authors are encouraged to rewrite the sentence for clarity.

**Response**: This sentence has now moved due to other amendments but has now been rewritten:

*“Expanding on the above concept, and citing the work of Porter (2,10), Winkelman (11) highlighted the impact of including a directional component in a cue to enhance motor performance.”*

**Reviewer**: Paragraph 3, Sentence 3-4: Authors need to include a reference for these claims.

**Response**: The relevant references have now been added.

**Reviewer**: Paragraph 4, Sentence 2: GC3

**Response**: As per our previous response (GC3), we have performed a general review of the writing style.

**Reviewer**: Paragraph 4: The authors are encouraged to either define analogies or metaphors. Also, if they are the same, stick to one term and use it throughout the manuscript. Authors are also encouraged to indicate how they can be different to instructions/AF.

**Response**: We have now added a definition (Capio et al., 2020) that indicates the difference to a normal cue.

*“An analogy is a coaching instruction that conceals biomechanical cues within spoken words. It differs from a conventional instruction in that it conveys the key elements of a given movement without the need to specific reference those same elements (17).”*

We now use only the terms ‘analogy’ and ‘analogies’ throughout.

**Reviewer**: Paragraph 4, Last Sentence: The authors are encouraged to rewire the sentence for clarity. A distal focus is done by focusing distally from the body, not by it simply being an EC or an analogy. For example, throwing a dart and focusing on the dart and or board are both ECs, but one is more distal than the other. Similarly, focusing on throwing the dart quickly like a gunshot or arrow, are both analogies, but do not give proximal or distal information.

**Response**: Agreed – we have now removed the term ‘distal’ as it is somewhat redundant here.

**Reviewer**: Paragraph 5, Sentence 2: Change “our” to “the authors” as it changes it from a subjective (2nd person) perspective to an objective (3rd person) perspective.

**Response**: We have now changed this.

**Reviewer**: Paragraph 5: The authors are encouraged to change the names of the conditions to reflect the specificity of the attentional focus, i.e. the “directional component” is drawing the attention externally in both ‘toward’ and ‘away’ conditions.

**Response**: We have now added the term ‘external’ here to differentiate.

**Reviewer**: Paragraph 5, Sentence 4-5: GC3

**Response**: In accordance with other changes, these sentences have been revised.

**Methods**

**Reviewer**: Paragraph 1: GC3. Authors are also recommended to include the total amount of sprints and jumps performed per participant per testing session.

**Response**: We have added this information.

**Reviewer**: Paragraph 2: The authors are encouraged to be more explicit as to the data processing. Currently, it is interpreted that a comparison across all the populations and measures was used to increase statistical power. However, these measures are open to an increase in error with the differences across training experiences, surfaces, apparatus, and matched-controlled cues. The mean of a highly trained group will be different than a moderately or poorly trained group, why are these means collated together and not interpreted separately?

**Response**: Thank you for this comment – we perhaps did not provide enough information at this stage of the paper. We did in fact also separately analyse all of the data from the different centres we just hadn’t indicated it here as we should have. It was an oversight as this constitutes 50% of our analyses for this paper and in line with a similar comment from Reviewer #1, we have added the following information:

*“We combined this approach with a within-country repeated measures analysis to determine the effect of the various coaching cues on performance at each of the six centres involved in the study. The design of the research can be viewed in Figure 1 below.”*

To come back to the reviewer’s original approach, we also appreciate the increase in error that accumulates across the various centres we used within this research. That is why we used a random effects analysis and standardised mean difference to offset these effects. Though they can not be entirely minimised, we felt *a priori* that this would still represent a logical approach given the lack of investigations in this population and felt that the internal meta-analytical approach could expedite knowledge in the area. We acknowledge the limitations both here and in the paper itself but combined with the repeated-measures analysis that compared the coaching cues at each of the venues, we also feel that this provides a strong, valuable and interesting two-pronged analysis that can be of use to practitioners in particular.

**Reviewer**: Paragraph 3: The authors are encouraged to rewrite the paragraph for clarity. The notion that the control cues provided no attentional component is false. The attention was to either jump HIGH or sprint FAST. No attentional component would simply be, “perform a jump” or “perform a sprint”. The current control conditions are of neutral focus, as they do not direct the attention internally or externally, but still direct the attention of the athlete.

**Response**: We appreciate the reviewer’s stance on this and have now deleted the line in question.

**Reviewer**: Paragraph 4 (Warm-Up): The authors are encouraged to describe if any feedback, i.e. cues or AF was given during the warm-up.

**Response**: No cues were provided during the warm up and we have now indicated this.

**Reviewer**: Paragraph 5 (Jumps): The authors are encouraged to rewrite the paragraph for clarity. Is this a countermovement jump? It can be inferred, but authors are encouraged to explicitly tell the reader what type of jump was completed.

**Response**: Yes, this was a vertical countermovement jump and we have now added/edited the text in this paragraph for clarity.

**Reviewer**: Similarly, the order in which ‘cues’, ‘instructions’ or ‘feedback’ was given is confusing. It seems that “jump as high as you can” (which is the control condition) was a higher dosage than any other condition. Why?

**Response**: We are unsure as to why the reviewer has concluded that the control condition was given in a higher dosage. As already indicated in the methods, each cue was given twice so all were administered an equal number of times:

*“There were five different cues for the sprints and five different cues for the jumps meaning each participant received each cue twice.”*

If the reviewer would be willing to provide further guidance on where this point of confusion is in the paper, we would be happy to revisit it.

**Reviewer**: Were these jumps performed Akimbo? Why or why not?

**Response**: Yes, they were performed akimbo to standardise execution across the various centres. We have now added this information to the paper:

*“For the test, the arms were positioned akimbo (i.e. with the hands on the hips and the elbows turned outward) and the feet positioned approximately shoulder width, at a distance comfortable for the participant.”*

**Reviewer**: How was the data then processed? Best trial on what measure? An average across two jumps? The ‘highest’ of the two trials is unclear, based on what measure, jump height?

**Response**: We have now added this information for clarity:

*“each participant’s best effort (i.e. highest jump in cm) out of two trials was used in the analysis”*

**Reviewer**: Paragraph 6 (Sprints): The authors are encouraged to rewrite the paragraph for clarity. Similar to the jumps paragraph, the order in which ‘cues’, ‘instructions’ or ‘feedback’ was given is confusing. It seems that the participants had to focus on a lot of information prior to performing the sprint, however, the ‘control’ condition relevant information was constantly reinforced throughout each condition?

**Response**: We acknowledge that there was quite a bit of information provided to the participants prior to the sprints, however, this was in an effort to stay in line with similar published studies in the area. We replicated the protocol of Winkelman et al. who we consider to have done very good and rigorous work in this area and so we wanted to align our methods with theirs. On the amount of information provided, this was not before every sprint but before the first one only and alongside a coach’s demonstration that was provided collectively, not individually (and was not repeated). The only information provided prior to the sprint was the specific cue as randomly allocated. We have now clarified and reworded this paragraph to address these issues.

**Results**

**Reviewer**: The results are reported succinctly and clearly.

**Response**: Thank you for this comment. We have added some additional detail based on a request from reviewer #1 though we believe the clarity is still retained.

**Discussion**

**Reviewer**: Paragraph 1, Sentence 3-4: The authors are encouraged to combine these sentences to increase the clarity and reduce leading the reader to believe more evidence than a very specific finding is supporting evidence.

**Response**: We have added additional detail here and rewritten the paragraph for greater clarity.

**Reviewer**: Paragraph 2: The authors are encouraged to include the maturity levels of the participants to strengthen this discussion point. The comprehension of the verbal cue is important and does develop with an increase in maturity. However, it is unclear what the maturity age of the participants is as only chronological age is included. Were the participants, pre-, circa-, or post-peak height velocity?

**Response**: We appreciate this point from the reviewer. Unfortunately, we did not collect biological maturity data as it would have been very difficult given what was a highly arduous and finely balanced data collection process on each testing day. Based on chronological age as a very loose indicator of “likely” biological maturity status, we did appear to have children in the pre-, mid- and post-peak height velocity stages of maturation. However, in this paragraph, we talk about neurocognitive capacities which do not develop linearly with physical capacities during growth and maturation and so the use of Mirwald’s maturity offset, Khamis and Roche’s predicted adult height or Tanner staging would have had limited use in supporting the points we were attempting to make here.

**Reviewer**: Paragraph 2, Sentence 4-5: The authors are encouraged to rewrite the sentences for clarity. Sentence 4 is also a run-on.

**Response**: We have split sentence 4 into two thus eliminating the run-on sentence. We have also split sentence 5 for clarity.

**Reviewer**: Paragraph 3, Sentence 1-3: The authors are encouraged to put this content in the introduction as it introduces the relevance of the constrained action hypothesis to the reader.

**Response**: This information now also appears in the introduction in a similar guise give that both reviewers had requested changes that necessitated this. We felt a reiteration of this information was necessary in the discussion as we will potentially be explaining a set of unexpected results to somewhat naïve readers in many cases, and so wanted to re-include this information to provide context.

**Reviewer**: Paragraph 3: The notion that the constrained action hypothesis does not work in children is not supported by the literature. See the following:

Tse ACY, van Ginneken WF. Children’s conscious control propensity moderates the role of attentional focus in motor skill acquisition. Psychol Sport Exerc 31: 35–39, 2017.

Chow JY, Koh M, Davids K, Button C, Rein R. Effects of different instructional constraints on task performance and emergence of coordination in children. Eur J Sport Sci 14: 224–232, 2014.

Prapavessis H, McNair PJ, Anderson K, Hohepa M. Decreasing landing forces in children: The effect of instructions. J Orthop Sports Phys Ther 33: 204–207, 2003.

**Response**: We agree with the reviewer that our language was far too strong here and we have now moderated it according to the reviewer’s recommendations. We also acknowledge the literature cited by the reviewer and agree that the constrained action hypothesis can and does hold in children. At the same time we must acknowledge the strength of our own results and so we believe the rewriting of the text in question now offers a better balance between this and the extant literature:

*“Based on the extant evidence, these concepts seem to hold in adults (37) yet according to our results at least, the predicted outcomes of constrained action hypothesis could be impeded in a youth population. Why this is the case is not entirely clear; however, there could be a practical explanation for our finding that has previously been raised by Maxwell and Masters (38). These authors demonstrated that when asked to perform a balance task, performers who were provided with an internal focus of attention had switched to an external focus of attention once they executed the task.”*

**Reviewer**: Paragraph 4: The authors are considering the participants as naïve participants, but some have training ages of 4+ years. Could it instead of an effect of training age rather than all young participants being considered naïve? Young participants have been shown to elicit high levels of force and power production as long as they are trained appropriately. See:

Lesinski M, Prieske O, Granacher U. Effects and dose-response relationships of resistance training on physical performance in youth athletes: A systematic review and meta-analysis. Br J Sports Med 50: 781–795, 2016.

**Response**: Yes, the reviewer makes a good point and we do acknowledge the fact that young people can indeed exert high levels of force. To clarify, we don’t necessarily see ‘training age’ as the same thing as ‘life experience’. For example, we have a number of active coaches on our research team for this study and several of them have suggested that even their more experienced athletes can be naïve in terms of their life experiences, their ability to follow instructions and their understanding of what coach is asking. Essentially, despite being experienced trainees, they remain “immature” for want of a better term. In many cases, an experienced athlete could have undertaken four years of training but never been exposed to analogies as a coaching tool and so they would remain naïve to their use in this context. We have now clarified the text here to avoid confusing our readers. For example, we now differentiate ‘experience’ from ‘life experience’ so as to provide further context to this paragraph.

**Reviewer**: Paragraph 5: The authors are eluding that the information may not have been relevant enough. However, Winkelman also eludes that when cues are compared, they should be matched-controlled (see Winkelman NC, Clark KP, Ryan LJ. Experience level influences the effect of attentional focus on sprint performance. Hum Mov Sci 52: 84–95, 2017.) Thus, it is possible that the neutral cue, which was reinforced throughout each condition across jump and sprint tests received more exposure and was also simpler and more similar to what a coach would say than the other ‘cues’ that were more instructions rather than a practical cue.

**Response**: We do agree that the neutral cue is simpler and more similar to what a coach would say and that in itself is very interesting as perhaps this is what coaches should be doing with young people rather than using internals, externals or analogies? Our evidence isn’t quite unequivocal enough to support that claim just yet but it is a potential avenue of future study. That aside, we are a little unsure as to what the reviewer means in this instance as the neutral cue was not delivered more times that the experimental ones. As now clarified in the method section, the neutral cue was delivered the same amount of times (i.e. twice) as every other cue so would not have been more reinforced with the participants. If we are misunderstanding the reviewer here, we would be happy to revisit the comment upon further advice.

One potential reason this confusion may have been caused was the way in which we described the internal meta-analytical analysis so we have added a clarifying sentence to the statistical analysis section of the methods:

*“The neutral control cue was compared separately against the ECs, ICs and two different ADCs resulting in four analyses for jumps and four for sprints (EC vs control, IC vs control, and ADCs (x2) vs. control).”*

**Reviewer**: Paragraph 6: See comments for paragraph 5 and reference that these ‘control’ cues still provided an attentional focus, to either jump HIGH or sprint FAST. Thus, they were not controls, but rather neutral focused cues. Limitations should rather point to the lack of matched cues, homogenous training age, language, or maturity level. All of which were described in the previous discussion paragraphs.

**Response**: We have now added the term neutral here – electing to call the terms ‘neutral control’ cues in this instance. From a research design perspective we believe it will help with readers’ understanding of our work if it is highlighted that these neutral cues were used as the control condition because they were the condition against which all the other cues were compared. In line with the reviewer’s recommendation, we now say the below and have made similar changes throughout the manuscript (including the methods):

*“The terms "jump as high as you can" and "sprint as fast as you can" were neutral cues that were used as control conditions against which the various ICs, ECs and ADCs were compared (25,26).”*

We also state:

*“There is a chance that this was because there is no established standard as to what constitutes a control cue meaning these neutral cues were just as effective as the experimental cues in driving performance in the study participants.”*

In relation to limitations, we have now added language and biological maturation to the text and had already referenced the fact that experience level was a potential limitation in this investigation.

**Conclusion**

**Reviewer**: The authors should consider re-writing the conclusion to include the considerations of the reviewer. The study is interesting, but the way the manuscript is written indicates that external cues do not provide benefit over internal or neutral cues in young populations. This notion contest previous research and should be given a deeper critical evaluation as to why.

**Response**: We have edited the conclusion to add some critical insight as to why the external cues did not result in enhanced performance. This possibly relates to life experience and the attentional capacity of youths relative to adults and we have used this as a basis to recommend future work in this area.
