# Supplementary material for: Do verbal coaching cues and analogies affect motor skill performance in youth populations?
Source: PLoS One. 2023 Mar 2;18(3):e0280201. doi: 10.1371/journal.pone.0280201 (PMC9980803; doi:10.1371/journal.pone.0280201)
Supplement: Attachment — Submitted filename: Response to reviewer.docx [file pone.0280201.s013.docx]

We would like to sincerely thank the reviewer once again for the time that they have taken in evaluating the revised manuscript and for the recommendations that were put forward. Below we have provided an itemised response to each comment. In the vast majority of cases we were able to incorporate the reviewer’s recommendations into the paper and we extend our thanks for their help in improving the quality of the article.

Regards

Jason

**Reviewer #2: Review of Manuscript: PONE-D-22-21775 Do verbal coaching cues and analogies affect motor skill performance in youth**

**populations?**

**Title: Do verbal coaching cues and analogies affect motor skill performance in youth**

**populations?**

**The authors have written a manuscript investigating cues of external (EC) or internal (IC) focus, with an additional condition of analogies with a directional component (ADC) on sprint and jump performance in youth performers. A control cue condition was also included.**

**The authors have addressed a majority of the comments. There are still a few general comments and specific comments that would strengthen the rigour and quality of the manuscript.**

Below are general comments for the author's consideration.

General Comments (GC):

**Reviewer #2: GC1: The tables are not fully legible as they are cut off in the submission.**

Response: This had to do with the portrait/landscape orientation of the pages which we have now remedied. The tables should now be fully viewable.

**Reviewer #2: GC1: GC2: The authors are encouraged to stop using “we” and “our” in the manuscript and maintain an objective (3rd person) writing style throughout.**

Response: This has been changed and edited in each instance in the manuscript with sentences amended according to the context.

**Reviewer #2: GC3: The discussion does little to discuss how CMJ jump height or 20m sprint performance has previously been affected by cueing interventions. Or why no benefits were observed in these particular measures. The authors are encouraged to discuss their measures.**

Response: We have now reviewed the discussion and added areas of text that align with the reviewer’s recommendation here.

Firstly, we have used the review of Barillas et al. (2021) to strengthen our choice of hypotheses in the discussion section. We feel this is a more efficient way to incorporate the type of research highlighted by the reviewer without having to cite all of the research studies in this area:

*“Previous research has demonstrated that coaching language, such as ECs, can have a positive effect on sprint and jump performance; however most evidence relates to adult rather than youth populations (Barillas et al., 2021). Accordingly, it was hypothesised that ECs, ICs and ADCs would be more effective than neutral control cues for enhancing jump and sprint performance, that ECs would be more effective than ICs and that ADCs would be more effective than both ECs and ICs.”*

We have also used cited the review article of Barillas et al. to reinforce the reviewer’s first point of this recommendation. We feel this is a more efficient way to summarise the literature in this case.

*“However, as youths develop as they age, their neurocognitive capacities, as well as their ability and willingness to follow instructions, can vary (Kushner et al. 2015). This means that when working with youths, the challenges of coaching may be different to those encountered when working with adults (Kushner et al. 2015). The results of the current study are indicative of this when compared to current literature which indicates that coaching techniques such as ECs result in enhanced sprint and jump performance in adults and youths, though evidence in the latter group is relatively scarce (Barillas et al., 2021).”*

The reasons the reviewer might have the impression that the underpinning mechanisms for results are not discussed in extensive depth is that we freely admit in the paper that we are not entirely sure why they occurred in this manner across some fairly heterogenous male youth populations. Quoting ourselves:

*“Based on the extant evidence, these concepts seem to hold in adults (35) yet according to the current results at least, the predicted outcomes of constrained action hypothesis could be impeded in certain youth populations in performance tests such as the vertical jump and 20 m sprint. Why this is the case is not entirely clear; however, there could be a practical explanation for this finding that has previously been raised by Maxwell and Masters (36).”*

We then go on to elaborate on the work of Maxwell and Masters citing youths’ possibly shorter attention span offsetting the effects of the cues”

*These authors demonstrated that when asked to perform a balance task, performers who were provided with an internal focus of attention had switched to an external focus once they executed the task. Comparative research (37) that has been carried out in children and adults is suggestive of a shorter span of attention in the former group. The reason for this could potentially be explained by the rate of cognitive development in children and adolescents whose frontal lobes continue to mature as they grow (37).*

However, in light of the relative lack of research in youths (also highlighted in the review by Barillas et al., 2021), particularly that which focuses on underpinning mechanisms, we have now also added text to the end of the discussion:

*“…….research should also investigate the potential underlying mechanisms that determine attentional focus during motor skill execution in youth performers”.*

Elsewhere, in the discussion we elaborate even further on our results, attempting to remain tethered to a comparison to the bulk of the research on this topic, which has been carried out in adults. We believe this keeps the discussion grounded and ensures that only valid comparisons are made. Accordingly, we state:

*“Given that there were no differences in performance when ECs and ICs were used, the results of this study could point to the potentially narrower frame of reference that youths possess in comparison to adults which could, in turn, can have a detrimental impact on their understanding of a given coaching cue (12). It has been argued that because younger people have fewer past life experiences than adults, they could be classified as ‘naïve perceivers’ (40). Accordingly, young peoples’ life experiences may not have developed to the extent that they can contextualise instructions in the same way that adults do, particularly if those instructions are accompanied by an analogy that lacks context in terms of their understanding of a particular coaching cue. Moreover, whilst adults have been shown to focus on relevant cues only, children focus on both relevant and irrelevant cues and this could potentially impact on the level of attention they devote to a specific instruction (40).*

The above point is further elaborated on with input from the reviewer (please see below) but we must point out that we do feel we have discussed the relevant parts our findings sufficiently and in line with the extant evidence. Moreover, we must be careful in assuming too much with our results. Elsewhere in this review, the reviewer has suggested that we may have been overly speculative – an assertion that we are in agreement with. We now feel we have put forward well-reasoned arguments as to why no benefits were observed in the measures and with the reviewer’s assistance elsewhere in the discussion (exemplified by our below responses) have improved the paper substantially. On this basis, we have tried to strike a balance between the reviewer’s suggestion here and our own interpretation of our results and the reasons for them. We hope the reviewer can appreciate our perspective on this and there are some very important changes that we do implement as detailed below.

**Specific Comments:**

**Introduction**

**Reviewer #2: Line 100: Authors are encouraged to include ‘might’ or ‘may’ between ‘therefore’ and ‘not’.**

Response: Now edited. The sentence now reads:

*“Accordingly, coaches therefore might not be using optimal methods when attempting to drive motor learning in these populations.”*

**Reviewer #2: Line 111-122: To strengthen the evidenced based practice angle of the paper, authors are also encouraged to review and include the following citations in this paragraph:**

**Radnor JM, Moeskops S, Morris SJ, Mathews TA, Kumar NT, Pullen BJ, Meyers RW, Pedley JS, Gould ZI, Oliver JL, and Lloyd RS. Developing Athletic Motor Skill Competencies in Youth. Strength Cond J 42: 54-70, 2020.**

**Kushner AM, Kiefer AW, Lesnick S, Faigenbaum AD, Kashikar-Zuck S, and Myer GD. Training the developing brain part II: cognitive considerations for youth instruction and feedback. Curr Sports Med Rep 14: 235-243, 2015.**

**Both articles indicate that analogies should be prioritised when working with younger or less trained athletes. Particularly due to the limited abstract processing skills that will develop throughout maturation and mostly in adolescents. This better describes the mechanism at work which is inferred by Fasold et al.**

Response: Thank you for this recommendation and the associated references. We have now consulted both and included each in an amendment to this sentence which now reads as follows in line with the reviewer’s recommendation:

*“Recently, Fasold et al. (2020) found that children exhibited improved performance in handball skills when coaching cues were delivered in an analogical format. Indeed, it has been suggested that this method of coaching delivery should be prioritised when working with young athletes on the basis that it can improve information processing by enhancing the recall of instructions, thus making those instructions more relatable to the task to which they specifically refer (Kushner et al., 2015; Radnor et al., 2020).”*

**Reviewer #2: Line 126: Authors are encouraged to either maintain ‘toward’ and ‘away’ in quotations or no quotations. Currently, ‘towards’ is in “” whereas ‘away’ is not.**

Response: We have now amended this to read ‘toward’ and ‘away’ for consistency’

**Reviewer #2: Line 129-132: Analogies combined with ECs are likely not a new tool, but rather the evidence to support its use is new. The authors are encouraged to change the wording to highlight the novelty of the understanding, not the novelty of the practice. As previous literature recommends using simpler language, analogies, and metaphors, but based on cognitive limitations and not quantified changes.**

Response: We have now modified this sentence to reflect the reviewer’s recommendations:

*“This could have important implications for coaching and learning as the combination of ECs and analogies could represent a previously known, yet untested tool, for coaches that could enhance the contextual understanding of a performer and, by extension, the learning of key movement skills.”*

**Methods**

**Reviewer #2: Line 201-202: As per the comment where the control cue was given more exposure than the other cues, the reviewer mistakenly read the original paragraph to read that participants were reminded to jump as high as they can with each jump. However, in the new iteration, it is now referenced here that “Prior to the jumps taking place, each participant was individually requested to ‘jump as high as you can in the remaining ten jumps’”. It is unclear, but would an athlete have experienced this format:**

**1. Jump as high as you can in the remaining ten jumps**

**2. EC / IC / ADC / Control**

**3. Jump 1**

**4. EC / IC / ADC / Control**

**5. Jump 2**

**6. EC / IC / ADC / Control**

**7. Jump 3**

**8. Etc…**

Response: Again here, we were perhaps not clear enough in how we described the experimental protocol but to answer the reviewer’s question in short: no, the participants would not have experienced this format of instruction. In the format put forward by the reviewer, it appears that each cue is delivered for each jump. This was not the case. There were 10 cues and each was associated with just 1 jump (so 10 jumps in total, 1 cue per jump). A full jump protocol would have looked like this:

- Before any cue was delivered or any jump/sprint took place, the following was communicated to the participants about the protocol in general (i.e. not in relation to one specific jump or sprint):
  - “Please jump as high as you can in the remaining ten jumps. Prior to each jump you will be given a specific coaching cue. Focus as hard as you can on this cue during the jump”
- They then would have been given a random jump scheme that may have looked like the below, let’s say it was ‘scheme 9’ (the order within which the cues were also randomised, ensuring double randomisation of both schemes and cues):
- Jump 1: “jump as if you are trying to catch a ball overhead at its highest point"
  - Athlete then jumps
- Jump 2: “jump as if you are trying to catch a ball overhead at its highest point"
  - Athlete then jumps (the randomisation process is what accounted for jump cues 1 and 2 being identical in ‘scheme 9’ but this pattern does not continue as seen below).
- Jump 3: “jump as if the ground is suddenly hot and you have to get off it as quick as possible"
  - Athlete then jumps
- Jump 4: “as you jump, focus on extending your legs"
  - Athlete then jumps
- Jump 5: "jump as high as you can" (CONTROL CUE)
  - Athlete then jumps
- Jump 6: “as you jump, focus on extending your legs”
  - Athlete then jumps
- Jump 7: “as you jump, focus on pushing the ground away”
  - Athlete then jumps
- Jump 8: "jump as high as you can" (CONTROL CUE)
  - Athlete then jumps
- Jump 9: “as you jump, focus on pushing the ground away”
  - Athlete then jumps
- Jump 10: “jump as if the ground is suddenly hot and you have to get off it as quick as possible”
  - Athlete then jumps

On the basis of the above confusion, we have now amended the wording to state “Prior to any jumps taking place”………as below:

“Prior to any jumps taking place, each participant was individually requested to “jump as high as you can in the remaining ten jumps”

We have also done this for the 20-m sprints.

**Reviewer #2: If so, the neutral cue of 'Jump as high as you can' is still given a net 1 more times than the previous (this is similar to the delivery of cues for the Sprint task too).**

Response: We can see what the reviewer saying here, however, we do feel that the effect of this is almost entirely negligible, a point we hope is supported by the elongated format we have shown above which outlines a full example order in which the cues were given. For example, because of our double randomisation process, the delivery of the first general instruction can theoretically be 9 or 10 specific cues removed from the delivery of the actual control cue. The participants in the study were very well aware of which words were simply preparatory in nature and which words constituted the actual control cue to perform in response to. All were well briefed prior to the study being undertaken and the protocol was carried out with precision at each of our centres.

**Reviewer #2: Similarly, it is delivered in a more natural format. To be a control for the IC and EC, it should have been delivered as “As you jump, focus on getting as high as you can” rather than “Jump as high as you can”. Or alternatively, the IC be, “Extend your legs (powerfully/forcefully/quickly)” and the EC be, “Push the ground away (powerfully/forcefully/quickly)”. This mismatched control cue should be highlighted as a limitation. However, the reviewer respects that this area of research is still being defined and parameters are still being understood. Interestingly, the results point to the control cue providing the most benefit, and it’s the cue that would be most commonly used in practice and reduces cognitive processing strain with less irrelevant information.**

Response: Yes, this is a good point and we can see what the reviewer is saying here. Despite this, we don’t think it would have affected the results to any great extent (particularly given that most cues seemed to yield similar performances regardless of cue, task, place or language with a few exceptions). In agreement with the reviewer, we have now highlighted this as a limitation and, indeed, will take it on board for future work we do in this area. The limitation, alongside the pre-existing text now reads:

*“Similarly, the ECs and ICs required the participants to retain a specific focus for performance whereas the neutral cues simply requested maximal performance. This small differential could impact on an individual’s understanding of a particular cue and though it was deliberate in nature, researchers must work to standardise cues across various tasks to ensure the most effective form of communication.”*

Just to give a little insight on the issue, we deliberately based the control cue on previous research in this area (25,26) and we felt the cue itself needed to be as specific to the task possible. For example, “sprinting as fast as one can” is an entirely clear and possible task to achieve – the participant merely performs as best they can. Similarly, focusing on “driving the ground back” is entirely possible but literally doing it is a different matter altogether! We admit that we are delving into semantics on this point but we feel there are multiple ways in which words and language can either differ or be similar and it is a difficult balance for coaches and researchers alike.

This is reinforced by our pre-existing recommendation for more work in this area:

*“Accordingly, alternative cues with different compositions, and in other languages, should be tested to examine the most effective coaching terms to enhance performance in young individuals.”*

We hope the above serves as a rationale as to how we approached this work.

**Results**

**Reviewer #2: The authors have kept the succinctness of the results with the additional text. It illustrates the absence of an effect across intervention cues.**

Response: Thank you again for your acknowledgement of this.

**Discussion**

**Reviewer #2: Line 319-321: Authors are encouraged to include Kushner et al (2015) to support this statement.**

Response: We have now added the Kushner reference to this sentence.

**Reviewer #2: Line 339-341 & Line 348 -351: The authors make a general statement that their results showcase the constrained action hypothesis may be impeded by youth populations. However, this is a very general statement and is only true for naïve youth populations with measures of akimbo CMJ jump height and 20m sprint time. The authors are encouraged to be more specific as research currently exists that young (~11-year-olds) and old (~16-year-olds) youth athletes benefit from the constrained action hypothesis (see references):**

**Barillas, S. R., Oliver, J. L., Lloyd, R. S., & Pedley, J. S. (2022). Kinetic Responses to External Cues Are Specific to Both the Type of Cue and Type of Exercise in Adolescent Athletes. Journal of strength and conditioning research. (published ahead of print)**

**Oliver, J. L., Barillas, S. R., Lloyd, R. S., Moore, I., & Pedley, J. (2021). External Cueing Influences Drop Jump Performance in Trained Young Soccer Players. Journal of strength and conditioning research, 35(6), 1700–1706.**

Response: In relation to lines 339-341, we have now been more specific with the language to reflect the recommendations of the reviewer. The lines are now rewritten as such:

*“Based on the extant evidence, these concepts seem to hold in adults (37) yet according to the current results at least, the predicted outcomes of constrained action hypothesis could be impeded in certain youth populations in performance tests such as the vertical jump and 20 m sprint.”*

In relation to lines 348-351, we have also amended the wording to state that this may apply to certain naïve youth groups and we have now cited Barillas et al. (2022) and Oliver et al. (2021) as evidence that counteracts that which we present in the current study. We hope this gives a more rounded view of the literature as it currently stands and acknowledges the important work of others in this growing domain of research:

“Whilst concepts such as the constrained action hypothesis might serve as an effective model for motor performance and learning in adults, an alternative approach could be more appropriate in certain (perhaps naïve) youth groups due to the aforementioned factors, though evidence to the contrary does exist (Barillas et al., 2022; Oliver et al., 2021).”

**Reviewer #2: Line 355-368: The paragraph seems to continue the argument that these young participants were unable to realise the benefits of the constrained action hypothesis. However, the argument seems to put the weighting on the participants, as they were unable to focus on only the relevant information. Rather, it could be argued that they were unable to realise the benefits of the constrained action hypothesis because they were given irrelevant information, thus blunting a potentially beneficial effect. This is supported by the neutral cue receiving the most benefit in CMJ jump height. The paragraph seems appropriate to discuss this limitation, rather it implies that only adults can benefit from external cues. Again, see the previous two references. It could be argued that if they were more trained and had more exposure to the cue to no longer be ‘naïve’ then maybe they would receive a benefit. However, in the current state, the paragraph does not read this way.**

Response: This is a very good point by the reviewer and very much understand the angle they come from. Accordingly, we have now added text (and the Barillas et al. 2022 reference) to this paragraph in line with the recommendation of the reviewer which reads as such:

*“On the contrary, it is important to consider that if a young individual achieves a sufficient volume and quality of training, it is possible that they would no longer be considered to be a naïve perceiver and so may respond more readily to ADCs or ECs when provided by a coach (Barillas et al., 2022)”*

In addition to this, we have also deleted some of the text relating to adults so as to soften our perhaps overly-stated point here. The following sentence has now been taken out of the manuscript:

*“On this basis, if presented with an ADC cue that is intended to elicit a specific motor response, that response might be more likely to be observed in an adult, who might have a better contextual understanding of the scenario that is verbally described by the coach.”*

**Reviewer #2: Line 370-386: This paragraph is discussing how ADCs might be an effective way of driving motor performance. However, the only two measures included in the study were CMJ jump height and 20m sprint time. What measures are included in the study to come to the conclusion that ADCs might improve motor performance? If there are measures of motor performance or perception of the cues, please include them in the methodology and results. If not, this paragraph needs to be removed as it would then be entirely speculative.**

Response: We can see what the reviewer is saying here and the problem may have been caused by our somewhat inaccurate (perhaps even verbose?!) way of expressing ourselves in this paragraph. First off to address the reviewer’s concerns, we have now amended the terms here, replacing ‘motor performance’ with the specific names of the tests that were actually conducted, those being the CMJ and the 20 m sprint. As an example, we now state instead:

*“We did observe limited evidence that ADCs might be an effective way of driving improved performance in sprinting and jumping in young individuals.”*

The above is a factually true statement that is reflected by our results and so we believe it is worth discussing in the manuscript. Also, in line with the reviewer’s recommendation, we have now deleted part of the paragraph in question. We are in agreement that the below sentences are too speculative in nature and are probably not within the scope of the study. Upon re-reading, we do agree that this text is misplaced:

*“The practice of storytelling to increase both sport and exercise participation in youths has previously been utilised with characters from Disney movies being used as a prism through which children can contextualise their experiences in physical activity (19–21). In a similar vein, it appears that ADCs can be used to achieve a greater level of engagement in youths.”*

Coming back to our above observation that our results are factual and thus must be discussed in the discussion section, we do not necessarily agree 100% that the paragraph is too speculative and that with the above amendments it is now more in line with the current literature in this domain. Indeed, even if it were speculative, we do believe that there is room for such reasoned speculation in a discussion section so as to offer potential explanations for the observed results and potential future avenues of investigation. This happens all the time in strength and conditioning based studies which include a marker of performance, but perhaps no marker of potential mechanism(s).

The new paragraph, pasted below, does not contain a single statement that is not supported either by our own current results or the results and/or opinions of other prominent authors in this field. The newly edited paragraph does not use language or terms that are unequivocal in nature, meaning we are open to questioning on each claim that we make (as we are through this current peer-review process). We feel we have avoided any hyperbolic claims and have adhered to common practice and evidence-based results throughout the piece. Perhaps more importantly however, what remains of the previous paragraph can be of use to coaches. As coaches ourselves, we wish to engage with research that not only speaks to the academic community, but also the coaching community. In this paper we seek to do this and so the promotion of sound coaching practice is central to the discussion of our results. We do hope the reviewer can appreciate our stance on this and the new paragraph now reads as such:

*“We did observe limited evidence that ADCs might be an effective way of driving improved performance in sprinting and jumping in young individuals. The use of analogies in coaching youths may serve as a more relatable model of communication that facilitates a better understanding of a coach’s cue than the use of traditional biomechanical terminology (35). In this way, the evocative language of instructing a young athlete to “take off like a rocket” (6) could be preferable if it results in a better contextual understanding than a cue relating to the movement and angles of specific limbs. However, where ADCs could potentially have drawbacks is in relation to the kinetic and kinematic characteristics of a given movement. For example, Winkelman states that if the information verbalised by a coach is not related to the task-relevant characteristics of a given motor skill, a cue may be less effective (11). Moreover, as an analogy can contain several different pieces of information in a single cue, it could have a negative effect on working memory during performance (41). Accordingly, the relevance of the information provided, relative to the action being performed, appears to be vital in driving sprint and jump performance in young individuals.”*

**Reviewer #2: Line 388-403: The limitation paragraph briefly discusses that the lack of standardization within neutral/control cues could have led to the observed benefit. Rather, the current evidence in the field points to the neutral control cue condition having less irrelevant information, delivered in a manner more akin to a way a coach would do in practice, and potentially highlighting an extra time at the beginning of each set of jumps or sprints. It is really hard to argue that these youth populations, all of whom play sports (soccer or rugby) aside from two school groups, would not be naïve to a 20m sprint. This paragraph should take the lack of standardisation argument towards future research using content-matched cues.**

Response: We have already added to this limitations paragraph based on other recommendations of the reviewer but we have now further strengthened it based on the comments put forward by them here. Accordingly, we have now acknowledged the importance in considering the combination of the cue type and the experience level of the performer:

*“An important consideration here is for researchers to compare the effects of content-matched cues in both naïve and non-naïve populations alike as the results could be different in each based on different combinations of the type of cue delivered and the level of experience of the performer”.*

**Reviewer #2: Line 400-403: These lines of future research indicate that there may potentially be maturity and sex differences. However, this notion lacks any supporting rationalisation. The authors are encouraged to either justify why there would be a maturity or sex difference that could have interacted with the findings of the current study or remove these two lines.**

Response: We have now deleted these lines in line with the reviewer’s recommendation.

**Conclusion**

**Reviewer #2: Line 405-406: Again, this is too general, a positive effect on CMJ jump height or 20m sprint time. It is not a valid statement to base the entirety of a youth’s motor skill performance on these two measures.**

Response: In line with this comment we have now amended this sentence to reflect the reviewer’s recommendation:

*“Our results did not generally indicate that the type of experimental cue used had a positive effect on performance tests such as the vertical jump and 20 m sprint in youths.”*

**Reviewer #2: Line 408-409: Again, it is unclear how ADCs were more effective than ECs and ICs, and in what way. What were the measurements? What was the magnitude of the effect?**

Response: We appreciate this and have now added the detail of what the measurements were. The magnitude of the effect is already reported elsewhere in the paper so is not repeated here.

We have now edited the text to read:

*“There was no evidence to support the initial hypotheses that ECs, ICs and ADCs would be more effective at enhancing vertical jump and 20 m sprint performance than neutral control cues, or that ECs would be more effective than ICs for the same measures.”*

And just below that, we have added further detail:

*“There was however some limited evidence that ADCs are more effective than both ECs and ICs at enhancing 20 m sprint performance.”*

**Reviewer #2: Line 414-426: These two paragraphs seem to contradict each other. The Line 414 paragraph highlights that ECs and ADCs will only work in adults or more ‘developed’ youth participants; whereas, the Line 423 paragraph highlights that ECs and ADCs should still be used with youth populations. A conclusion that fits with what has been measured is that ECs, ICs, and ADCs do not seem to positively affect CMJ jump height or 20m sprint time. However, these findings should not deter practitioners from using ECs, ADCs, or ICs with youth populations as all cueing strategies help with the learning and performance of a motor skill.**

**Similarly, more research is needed to see how ECs, ICs, and ADCs affect the performance and learning of motor skills by including more motor skill tasks and extensive measures.**

Response: Thank you for this recommendation. We have incorporated virtually all of it into our conclusion, though have not pasted it here as it is a little lengthy for response document.
